# CyTOF as a suitable tool for stratification and monitoring of cancer patients

**DOI:** 10.1186/s12967-025-06764-0

**Published:** 2025-07-01

**Authors:** Barbara Seliger, Chiara Massa

**Affiliations:** 1 Medical Faculty, Martin-Luther-University Halle-Wittenberg, Magdeburger Str. 2, 06112 Halle (Saale), Germany; 2 Fraunhofer Institute for Cell Therapy and Immunology, 04103 Leipzig, Germany; 3 Institute of Translational Immunology, Brandenburg Medical School “Theodor Fontane”, Hochstr. 29, 14770 Brandenburg an der Havel, Germany; 4 Faculty of Health Sciences Brandenburg, Brandenburg Medical School “Theodor Fontane”, Brandenburg, Germany

**Keywords:** Mass cytometry, CyTOF, Tumor, Immunotherapies, Immunomonitoring, Blood, Tumor tissue, Biomarker

## Abstract

Despite the recent implementation of immunotherapies into clinical practice of various tumor types, the immunobiology of tumors and in particular the role and clinical relevance of the different immune cell populations and their function still remained unclear. Therefore, an in depth analysis of the complex landscape of immune cell populations and their soluble mediators in the peripheral blood and tumor microenvironment of cancer patients is urgently needed. Mass cytometry has revolutionized the immune phenotyping in particular in settings where simultaneous breadth and detailed characterization of the phenotype and function of immune cell (sub)populations with limited sample size is required, such as monitoring of patients’ response to immunotherapies. Since mass cytometry is a powerful multiplex approach to decipher tumor intrinsic and tumor extrinsic effects of tumor immunotherapies, this review summarizes the use of this technology for determination of the frequency and functional status of immune cell populations within the tumor and in the blood leading to the identification of intratumoral/peripheral immune signatures that might serve as biomarkers (i) for treatment response and/or failure, (ii) for the stratification of tumor patients or (iii) for the identification of novel therapeutic targets.

## Background

During the last two decades, different kinds of immunotherapies have revolutionized tumor therapy leading to durable clinical responses, but unfortunately only in a limited number of patients, ranging between 20 and 40% depending on the tumor histotype [1]. Thus, there is an urgent need for biomarkers indicating a favourable response as well as an early detection of disease progression under therapy, which would allow patients’ stratification to the optimal therapeutic strategy. Recently, a number of predictors of response to immune checkpoint (ICP) inhibitors (ICPi)—predominantly tissue-based—have been reported, such as the frequency of tumor-infiltrating lymphocytes (TILs), high expression of programmed death ligand 1 (PD-L1) in biopsies as well as microsatellite instability (MSI) and tumor mutational burden (TMB) [2–6]. However, these parameters are hampered by the small number of parameters analysed, the tumor heterogeneity, the high plasticity and dynamics of the interaction between malignant cells and components of the immune system and the limited longitudinal observation window, which is important to trace markers associated with response or resistance to ICPi therapy. Indeed, it has been demonstrated that the levels of circulating tumor DNA (ctDNA) [7] or subsets of γδ T cells [8] correlated with therapeutic efficacy. Non-invasive approaches, such as a blood draw, allow molecular analysis of circulating tumor cells [9] as well as an accurate delineation of the composition of peripheral blood mononuclear cell (PBMC) over time to dynamically monitor disease progression, treatment and prognosis [10], which can improve the prediction of responses to different types of immunotherapies, including ICPi and chimeric antigen receptor (CAR) cell therapies. In addition, blood profiling has numerous advantages over tissue-based evaluations, like absence of bleeding, minimised costs and low invasiveness, which allow multiple, repeated sample collection over time. The identification of relevant responding cell (sub)types will provide insights into the underlying immunological mechanisms of primary and acquired resistances of diverse immunotherapies [11, 12]. In this context, high dimensional single cell cytometry by time-of-flight (CyTOF) analysis, also termed mass cytometry, will be a most efficient strategy and will give novel information regarding the mechanisms of action of immunotherapies, accelerating the development of therapies with improved efficacy and safety profiles enabling clinicians to better predict and monitor patients.

### Features of mass cytometry (cytometry by time-of flight [CyTOF]) versus standard flow cytometry

Since many decades, conventional standard flow cytometry has been employed in the clinical routine for the diagnosis of haematopoietic malignancies as well as for evaluation of the immune status of patients with different diseases. Due to the high level of functional heterogeneity of immune cells with different polarisation (type1, type2, type17, regulatory), memory properties (naïve, effector, memory) and activation / exhaustion states, an increasing number of parameters has to be evaluated in parallel in order to perform an appropriate characterisation of immune cells based on phenotypical evaluation, an assay much easier to standardize and apply in clinical routine than “real” functional immune cell assays. 

**Fig. 1:**
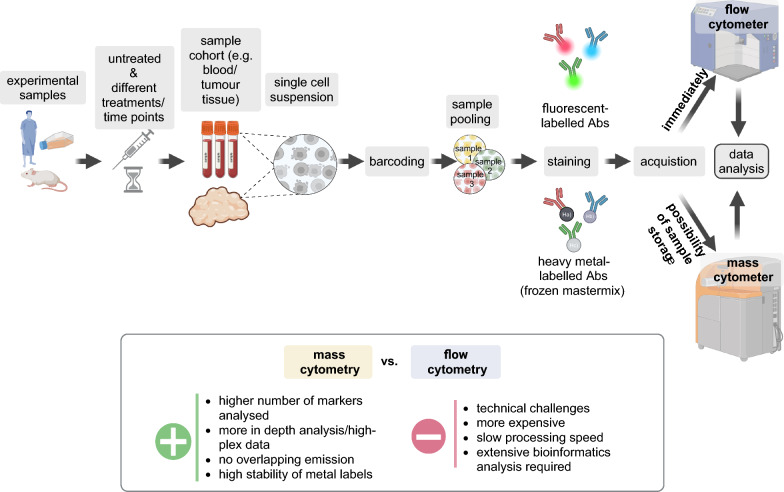
Comparison of standard flow- and mass- cytometry. Shown is the general workflow for flow/mass cytometry evaluation of samples derived from in vitro experiments, pre-clinical mouse models or patients as well as a pro–contra comparison. The figure was created with https://www.biorender.com/

Over the years, introduction of new lasers and additional fluorochromes has increased the number of parameters that can be acquired in parallel by standard flow cytometers to more than 25 [13]. The recent development of spectral flow cytometers has further expanded the number of markers that can be evaluated simultaneously up to 50 different parameters [14], but in practice a maximum of 40 antibodies (Abs) are measured due to the complexity of creating the staining panels. In order to avoid the problem of overlapping emission spectra of fluorescence-labelled Abs, cytometry has been combined with mass spectrometry giving rise to CyTOF or mass cytometry [15]. In this approach single cells suspensions are stained with non-radioactive, non-rare and non-biologically available metal isotopes instead of fluorochromes (Fig. 1). Labelled cells are then nebulized into ion clouds and the individual metal signals measured in successive “pushes” by a time-of-flight mass detector. Labelling of the cells prior to acquisition with DNA intercalators [16] allows to associate the different “pushes” to a cell, whereas cisplatin can function as a viability dye to discriminate dead cells [17]. Thus, even in the absence of the forward and side scatter parameters of flow cytometry, it is possible to correlate the signal intensity of each isotope with its specific Ab enabling the measurement of analyte levels within a cell [18].

Ab labelling has been initially performed with metals from the lanthanide group and next to commercially available pre-conjugated Ab, kits and protocols for custom labelling have been developed [19, 20]. Over the last years, the number of evaluable targets has increased due to the implementation of additional metals, such as cadmium [21], palladium [22], indium [22], platinum [23] and bismuth [24]. Moreover, Abs have been conjugated with nanoparticles containing silver [25] or tantalum oxide [26].

An advantage of metal versus fluorescence labels is their stability, which leads to the possibility to prepare a large master mix that can be frozen into aliquots, thus reducing batch variabilities among different acquisitions due to pipetting errors [27]. Similarly, already labelled samples can be stored at low temperature before being acquired without (excessive) signal loss [28, 29], thus making an acquisition also possible at a different site than the collection and staining centre, a common situation in many clinical trials. Analogous to flow cytometry, many studies have been performed to develop standard operation protocols (SOPs) for cell collection, staining and acquisition [30], with a particular focus on the best protocol to standardise sample acquisition for clinical trials ongoing in multiple locations and over a long time during and after therapy [31–33].

A major disadvantage of CyTOF is the high rate of cell loss during sample staining and acquisition and its slow processing speed with “suggested” acquisition rates of only 300 to 500 cells/second compared to over 10.000/s of most flow cytometers. In assays focusing on rare cells, experimental pipelines have been developed to introduce purification steps (by magnetic depletion or fluorescence sorting) before mass cytometry in order to enrich the target population in the sample and reduce acquisition time. For this purpose, protocols for double labelling of Ab for fluorescence and mass cytometry have also been generated [34, 35].

The high number of available “channels” to be detected also allows to bring barcoding of samples to another level with respect to flow cytometry. Indeed, already using a 6-choose-3 setting, in which each sample is labelled with 3 different metals chosen among 6 available ones, allows to combine 20 different samples in one tube, but with the increasing number of reporters available, protocols of 7-choose-3 and also 9-choose-3, which allow 35 or 126 samples to be combined together, respectively, have also been created [36, 37]. Due to barcoding, not only staining and acquisition variability among samples are reduced, but also the overall costs and acquisition time. Depending on the particular experimental setting, two main strategies for barcoding can be implemented, namely unspecific cell labelling by direct covalent conjugation of the metal to (intra)cellular proteins or specific staining with metal conjugated Ab. In the first setting, the basic protocol requires cell fixation and permeabilization [38, 39] thus posing a problem for subsequent staining with Ab specific for epitopes sensitive to fixation. To avoid this, both tellurium and selenium isotopes have been used to create more polar compounds which can be used for the direct staining of both fixed and live cells [40, 41], and which also allow the direct staining of organoids still growing within a matrigel matrix [36]. Otherwise, live cells can be specifically stained with Abs against widely expressed molecules, such as CD45 [42], if the main interest are immune cells, or beta 2-microglobulin with or without the sodium potassium ATP-ase subunit CD298, if also tumor and stromal cells have to be characterised [43]. To further enhance the number of barcoding, a nuclear barcoding based on two different platinum isotopes as DNA intercalators has been combined with the CD45 barcoding [44]. 

Recently, the experimental design for CyTOF concerning the limits of this technology has been reviewed [45]. Since not only donor-specific variations, but also sample sources as well as sample preparation, such as cell isolation, cell numbers, staining protocol, cell viability, fixation and Ab titrations, have an impact on the quality of cytometric analyses and results, SOPs should be developed for the processing and storage of samples to receive consistent and reproducible results through multiple sample sets [30]. Other important features are the selection of markers and the Ab panel design, which are critical for pairing of Abs with metal isotopes. It is noteworthy that despite much lower than in flow cytometry, some level of spill-over among the different “metal channel” is existing due to isotope impurity, possibility of metal oxidation during acquisition in solution as well as instrument abundance sensitivity. In order to simplify panel building, spill-over matrix for data compensation, like in standard flow cytometry, can be created [46].

One major limitation of mass cytometry, as of many other high-plex techniques, is the complex and large amount of data to be analysed and interpreted, which requires in depth computational expertise. Mass cytometry data are recorded in tables and formatted as FCS files, which could be analysed by available conventional flow cytometry software. Due to high dimensional data generated, different bioinformatics analysis tools have been developed, but their use require biological and statistical expertise [47, 48]. Next to this, the development of artificial intelligence (AI) approaches is required to enhance the accuracy in prediction of e.g. therapy response or resistance. Therefore, an automatization of the entire pipeline including data acquisition and bioinformatics analysis of the multiplex results is urgently required.

### Mass cytometry application in oncology

Mass cytometry analysis can be performed on whole blood, PBMCs, biological fluids, such as saliva, bronchial alveolar lavage and urine, and suspension of cells obtained from tissues. In 2009, mass cytometry was used for the first time for the comprehensive analysis of the immune cell function and activation in leukemia cell lines and leukemic patients’ samples [49]. Since then, this technology has been frequently applied in preclinical as well as clinical setting to better understand the interaction of tumor cells with their (immune) microenvironment and identify the mechanisms of response or resistance to treatment in order to optimize tumor therapy. Due to the high number of parameters, which can be analysed, mass cytometry has been implemented not only for surface phenotype, activation of signalling pathways and secretion of cytokines, but also to characterise epigenetic [50] as well as metabolic properties [51]. In addition, it can also be applied to perform “high resolution” receptor occupancy assay [52], which can help in determine the efficacy of therapeutic Ab directly in vivo*/*ex vivo.

In the following paragraphs, different studies implementing mass cytometry, frequently in parallel to other –omics-based techniques, such as RNA sequencing, but also low-plex flow cytometry, and their input in deepening the understanding of cancer biology and immunology and improve its clinical management, are presented.

### Use of mass cytometry for monitoring of the immune cell repertoire in blood and tissues of tumor patients

So far, various procedures have been developed by different groups for the in depth immunophenotyping of PBMC by high-dimensional CyTOF analysis leading to an increased knowledge of different immune cell (sub)populations, their heterogeneity and their dysfunction, such as e.g. T cells, natural killer (NK) cells and myeloid cells in tumor patients and healthy volunteers [53–55]. This will lead to the identification of novel immune cell subpopulations and of prognostic markers.

For example, in patients with acute myeloid leukemia (AML), an accumulation of unconventional CD56^−^ CD16^+^ NK cells, probably as a consequence of immune escape from innate immunity during AML progression, correlated with worse prognosis [56], thus suggesting the use of these molecules as prognostic markers.

In breast cancer patients disease progression was associated with an expansion of terminally differentiated Vδ2^+^ γδ T cells characterised by the expression of PD1 [57]. In another early study using the CyTOF approach, the peripheral immune landscape of peripheral blood from stage IV melanoma patients and age-matched healthy individuals was analysed [58]. This allowed to compile a detailed signature of different immune cell subpopulations in PBMC, which was associated with the patients’ survival.

Next to blood immunomonitoring, CyTOF protocols for the characterization of tumor-infiltrating cells were developed for human solid tumors and mouse models [59]. With the implementation of CyTOF, a huge diversity between different patients and within each individual not only between the blood and tumor immune cell infiltrate, but also between affected and non-affected tissues has been proved [60]. Simultaneous analysis of PBMC vs. tissue biomarkers from the same patients demonstrated that peripheral blood biomarkers are informative and clinically related to the outcome of tumor patients [53, 61] and thus might be used for determination the patient’s risk. On the other hand, it has been also demonstrated that the immune landscape of PBMC and tumors significantly differed and patient-specific immune changes should be taken into account for selection of treatment options.

Furthermore, a mass cytometric method was developed to track cell responses using a combination of T cell receptor (TCR) Vα and TCR Vβ chain-specific Abs and with Abs directed against T cell activation and differentiation markers [62], which allow to monitor changes in the TCR usage during adaptive (tumor) immune responses.

Next to the in depth analyses of immune cell subpopulations, also the release of cytokines can be monitored by mass cytometry. In myelofibrosis (MF) patients, monocytes were the main source of the constitutively overproduced cytokines responsible for the expansion of stem/progenitor cells [63]. IL8/CXCL8 was identified as the most upregulated cytokine associated with pro-survival and increased proliferation for myeloproliferative dysplastic syndromes (MDS) and AML [63].

### Use of mass cytometry to identify biomarkers for immunotherapy

Based on these features, CyTOF might be also used to identify novel biomarkers predicting therapy response. Indeed, there exists increasing evidence that a detailed analysis of T cells, in particular the characterisation of the heterogenic phenotype is of high relevance for T cell based immunotherapies [55].

- a. ICPi therapies

Since ICPi are known to produce a durable clinical response in only a limited number of tumor patients [1], it is important to identify biomarkers indicating not only “initial” favourable responses, but also early disease progression or the development of therapy resistances in order to optimally stratify patients. For this reason, mass cytometry has been highly implemented in the immunomonitoring of patients with different, mostly solid tumor histotypes undergoing different ICPi therapies.

From a technical viewpoint, a study compared different flow cytometric methods including mass cytometry for the most feasible and reliable method for accurately quantify the expression of the programmed death receptor 1 (PD1) on immune cells. This study confirmed the already known overlap between the epitope recognised by different therapeutic and detection Abs [64].

In patients with non-small cell lung cancer (NSCLC), the combined baseline levels of circulating classical monocytes, NK cells and ICOS^+^ CD4^+^ T cells were found to correlate with response to the anti-PD-1 ICPi pembrolizumab [65], resulting in the creation of a de-multiplexed, immune peripheral score based on those three cell types, which could be easily implemented in the clinical routine for stratifying patients to ICPi therapy. In another study with NSCLC patients undergoing pembrolizumab treatment, the longitudinal evaluation of the peripheral blood highlighted a much lower frequency of CD8^+^ T cell subtypes being also CD101^hi^ TIM3^+^, which represent exhausted T cells, in responder patients [66]. In melanoma patients, evaluation of blood before and after anti-PD1 therapy revealed that the baseline value of classical monocytes was predictive of response [67].

Investigation of the tumor microenvironment (TME) revealed that melanoma patients responding to the combination of ICPi against PD1 and CTLA-4 had an enhanced frequency of CD45RO^+^ EOMES^+^ T cells with an effector memory, but not terminally exhausted phenotype [68]. Comparison of peripheral blood and tumor infiltrate from melanoma patients treated with nivolumab alone or in combination with ipilimumab highlighted an enrichment of memory B cells within the tumor, but not in the peripheral blood of responder patients [69]. In sarcoma-bearing mice treated with PD1 and/or CTLA-4 ICPi a deep remodelling of both the lymphoid as well as the myeloid compartment has been identified [70]. Further evaluation was also done on resected lymph nodes (LN) from melanoma patients containing or lacking melanoma cells, confirming the co-presence of expanded activated, but exhausted T cells as well as suppressive mechanisms in the tumor-containing LN, suggesting to postpone LN resection after start of immunotherapy [71].

Similar studies have also been performed in order to identify biomarkers for the development of adverse events of toxicity to ICPi therapies. For this purpose, blood samples from patients with metastatic melanoma undergoing therapy has been deeply characterised by mass cytometry, as well as by RNA sequencing including T and B cell receptor clonotypes, thereby identifying high level of clonally diverse, effector memory CD4^+^ T cells to be correlated with the development of severe toxicity to ICPi [72].

Aside from the “standard” ICPi against PD1 and CTLA4, mass cytometry has also been implemented to identify regulatory mechanisms, which might impair patients´ immune response. For example, a detailed characterisation of the cells expressing the ICP molecule LAG3 within the infiltrate of different human solid tumors has been performed in order to identify markers for combinatorial therapy [73]. In a murine preclinical model, the MS4A4A receptor expressed by type 2 oriented tumor associated macrophages was evaluated as possible therapeutic target and the immune infiltrate of CT26 bearing mice was characterised by mass cytometry indicating a shift towards type 1 polarisation of the TME [74]. Similarly, deep evaluation of a preclinical glioblastoma model demonstrated reduced antigen presenting capabilities of DC within the brain, which might be a possible reason for the reduced responsiveness to ICPi in these tumors [75].

- b. CAR cell therapies

Chimeric antigen receptors (CARs) are synthetic fusion receptors, which were generated to redirect T cells to recognize and eliminate cancer cells that express tumor antigens [76]. Despite some success of this approach in particular in patients with B cell leukemia and lymphoma, challenges remain in optimizing their design, increasing their efficacy and their monitoring [76]. In this aspect, mass cytometry has played an important role, both at the preclinical and clinical level.

Due to its capability to evaluate many parameters in parallel and the development of protocols both for the sensitive detection of CAR^+^ cells [77] and for their phenotypical as well as functional characterisation [78, 79], mass cytometry has been implemented to determine the functional properties, persistence and metabolic fitness of T cells transfected with constructs implementing different costimulatory modules or homeostatic cytokines [80, 81]. Similarly, with the focus on additional effector cells for CAR transfection, construct / protocol optimisation has also been performed for NK cells [82, 83] and γδ T cells [80].

In the clinical setting, the infusion products and longitudinal blood samples before and during therapy have been in-depth characterised to identify biomarkers for patients´ response. With respect to the infusion products, higher expression of CD7, CXCR3 and NKG2D on CAR-T cells correlated with response of patients with diffuse large B cell lymphoma [84]. In patients with B cell acute lymphoblastic leukemia or large B cell lymphoma the presence of an NK cell-like subsets among the CAR-T cells associated with clinical outcome [85]. Finally, in patients with B cell lymphoma, who relapsed or were refractory to CAR-T therapy and were further treated with pembrolizumab, clinical responses were associated with a re-expansion of the CAR-T cells, which had an increased expression of cell activation and proliferation markers as well as a reduced sign of T cell exhaustion [86].

To sum up, the implementation of mass cytometry in the context of this novel treatment has the potential to assess the cytotoxicity and cytokine release following CAR-T cell therapy.

- c. Vaccination

Mass cytometry offers an expanding potential to decipher responses to infectious diseases and to vaccines by profiling protective immune responses post-infection and post-vaccination [87]. For example, combination of the inducible T cell costimulatory ligand (ICOS-L)-transduced B16 F10 cellular vaccine with CTLA4 blockade resulted in an enrichment of Th1 CD4^+^ T cells, effector CD8^+^ T cells and M1-like macrophages leading to an increased T cell function, immune cell infiltration and tumor rejection [88]. In the oncolytic setting, a live attenuated swine pseudorabies virus has been evaluated in a murine model for its potency at reprogramming the TME, alone and in combination to ICPi [89]. Similarly, vaccination using nanoparticles loaded with antigen-encoding mRNA molecules has been optimised with the inclusion of CpG adjuvant by evaluating the changes in the TME by mass cytometry [90]. In the clinical setting, mass cytometry immunomonitoring of glioblastoma patients receiving a peptide vaccine against a shared neoantigen derived from the H3.3K27 mutation revealed that expansion of antigen specific CD8^+^ T cells, independently of their functional status, associated with an increased patients’ survival [91].

- d. Hematopoietic stem cell (HSC) transplantation

In parallel to the evaluation of the reconstitution kinetics of the different immune subsets along time [37], mass cytometry has also been implemented in order to understand the effect of low dose IL-2 on the prevention of graft versus host disease in patients undergoing HSC [92].

### Use of mass cytometry for monitoring targeted therapies

The deepness of phenotypical as well as functional characterization allowed by mass cytometry was also implemented in the setting of targeted therapies, either at the preclinical level to identify the consequences of the inhibitor(s) and optimise their usage, or at the clinical setting to characterise resistant tumors and thus identify possible alternative/combinational therapies.

For example, mass cytometry was implemented to characterize the different signal pathways induced in blood from myeloproliferative neoplasia (MPN) patients as well as normal bone marrow (BM) samples by ex vivo treatment with different janus kinase (JAK) inhibitors [93]. In the patients´ specimens, the authors demonstrated a large population of CD14^+^ monocytes as a source of pro-inflammatory cytokines, which were differentially affected by distinct JAK inhibitors. Interestingly, these JAK inhibitors have both shared, but also unique profiles of pro-inflammatory cytokine suppression. This might be associated with a clinical activity of these inhibitors, which could also have an important impact on MPN disease progression [93]. Mass cytometry analysis of different neuroblastoma cell lines highlighted a heterogeneous upregulation of different pro-survival pathways upon P-selectin triggering, providing a mechanism for the reduced in vivo growth of neuroblastoma upon P-selectin inhibition in murine models [94].

In the opposite setting, mass cytometry has been implemented to profile the signalling pathways and phenotypical properties of drug resistant tumors in order to understand the underlying mechanism(s) of resistance and to possibly identify new targets for treating relapses. For example, the V561M mutation in the fibroblast growth factor receptor1 (FGFR1) provides a more mesenchymal phenotype to NSCLC cells, with higher vimentin expression and higher levels of phosphotyrosine, for example in STAT3, but also ERK and AKT, both at steady state and upon stimulation [95]. In a mouse model of breast cancer, inhibition of FGFR1 modulated cancer associated fibroblasts consequently allowing a higher infiltration of T cells [96]. Furthermore, human BC cell lines resistant to chemotherapy with paclitaxel and/or ruxolitinib have been evaluated by mass cytometry identifying a subpopulation of CD44^+^ CD24^−^ stem cells with enhanced JAK/STAT signalling in the resistant lines, which could be potentially therapeutically targeted [97].

A mass cytometry-based screening of in vitro responses to different drugs alone or in combination has been first established in the HeLA cell line, but then also applied to primary specimens from pediatric acute lymphoblastic leukemia [98]. As in many other settings, high levels of inter- and intra-tumor heterogeneity in responses have been identified. In a preclinical setting using a murine model of AML xenografts the efficacy of the combined treatment with sorafenib and the inhibitor of Wnt/β-catenin signalling pathway PRI-724 was determined and monitored by mass cytometry demonstrating a reduced number of human CD45^+^ cells and altered phosphorylation levels of many different pathways within these cells [99].

### Comparison of mass cytometry with other emerging high-dimensional technologies

Next to mass cytometry, other high dimensional technologies have been developed and applied to clinical samples in order to better understand the interaction between tumor cells and their microenvironment and thus improve patients´ diagnosis and stratification to the best possible therapeutic approach.

Similar to the developments in flow cytometry, standard immunohistochemistry (IHC) has been transformed into a high-plex technique either by performing sequential cycles of staining with fluorescently labeled primary or secondary Ab or by directly conjugating Ab to metal or oligonucleotides [100]. With respect to flow and mass cytometry, these techniques preserve the spatial context of cells within tissues thus allowing the analysis not only of the molecular properties of the different components of the TME, but also their spatial organization and possible interactions. Depending on the particular technique of multiplex IHC (mIHC), different amounts of tissue and thus number of cells, can be evaluated. Despite the algorithms for those high-plex histological evaluations are still limited, their potential for translational clinical application has been demonstrated by e.g. the identification of multiplex spatial cellular relationships in a large cohort of breast tumor samples, which improved histopathological classification [101]. In addition, imaging mass cytometry has been also implemented in different clinical trials, in order to help therapy selection [102, 103].

Whereas cytometry and imaging approaches determines the expression of a high, but limited number of markers, other -omics strategies, like genomics and transcriptomics, provide an unbiased evaluation of the cellular genotype and phenotype, but they miss the post-transcriptional and -translational changes in protein expression and functional status due to regulatory mechanisms, like micro-RNA and phosphorylation, respectively. With the increasing technical advances, single cell (sc-) and single nuclei (sn-)RNA-sequencing (RNAseq) are now possible and enable to assess even the whole transcriptome but, due to their costs, can evaluate a much more limited number of cells than (mass) cytometry. The latest development in transcriptome evaluation, namely spatial transcriptomic, enables (almost) transcriptome-wide profiling from tissue sections, thereby providing information for a broader number of cells and in association to their spatial distribution, but again cannot capture posttranslational modifications.

Thus, with respect to tissue evaluation with retention of spatial information, mIHC and spatial transcriptomics are complementary and synergistic technologies: while mIHC will be implemented for detailed protein phenotypic studies, spatial transcriptomics could be used for high-throughput transcriptomic exploration.

## Conclusions

In comparison to flow cytometry, mass cytometry significantly improved the in depth characterisation of the frequency and features of immune cell populations in the peripheral blood and tumor tissues of cancer patients, allowing also the identification of rare cell sub-populations, thus further highlighting the complexity of cancer and its heterogeneity among patients. While high dimensional mass cytometry is useful in revealing immunophenotypical characteristics of relevant cell types in diseases, standardization across centres and validation of results as well as data analysis in the clinical setting is challenging. Usage of anchor samples in combination with batch-normalisation algorithms can provide a way to obtain the required standardised and reliable data needed for clinical implementation [33]. Mass cytometry in combination with other –omics techniques identified different mechanisms responsible for response or resistance to various (immuno)therapies, which, upon de-multiplexing for easier translation into clinical routine, could be implemented as prognostic markers for patients´ outcome. For example, a composite score comprising classical monocytes, NK cells as well as ICOS^+^ CD4^+^ T cells was highly predictive for therapy efficacy and could be cost effectively determined in clinical routine via standard flow cytometry [65]. Thus, future research approaches should combine mass cytometry findings with data from other -omics technologies to e.g., uncover novel targets with therapeutic and prognostic impact and to better stratify patients in adequate therapeutic modes leading to improved personalized therapies.

## Data Availability

Not applicable.

## Abbreviations

Ab: Antibody
AI: Artificial intelligence
AML: Acute myeloid leukaemia
APC: Antigen presenting cells
BM: Bone marrow
CAR: Chimeric antigen receptor
CyTOF: Cytometry by time-of-flight
ctDNA: Circulating tumor DNA
FGFR1: Fibroblast growth factor receptor 1
HSC: Haematopoietic stem cell
ICOS: Inducible T cell costimulator
ICOS-L: Inducible T cell costimulatory ligand
ICP: Immune checkpoint
ICPi: Immune checkpoint inhibitors
IHC: Immunohistochemistry
JAK: Janus kinase
LN: Lymph node
MDS: Myeloproliferative dysplastic syndrome
MF: Myelofibrosis
mIHC: multiplex IHC
MPN: Myeloproliferative neoplasia
MSI: Microsatellite instability
NK: Natural killer
NSCLC: Non-small cell lung carcinoma
PBMC: Peripheral blood mononuclear cells
PD1: Programmed death receptor 1
PD-L1: Programmed death ligand 1
sc: single cell
SOPs: Standard operation procedures
sn: single nuclei
RNAseq: RNA sequencing
TCR: T cell receptor
TIL: Tumor-infiltrating lymphocytes
TMB: Tumor mutational burden
TME: Tumor microenvironment
